# Transmitted drug resistance in women with intrapartum HIV-1 diagnosis: a pilot epidemiological survey in Buenos Aires, Argentina

**DOI:** 10.7448/IAS.17.4.19704

**Published:** 2014-11-02

**Authors:** Diego Cecchini, Ines Zapiola, Silvina Fernandez Giuliano, Marina Martinez, Claudia Rodriguez, Maria Belen Bouzas

**Affiliations:** 1 Infectious Diseases Unit, Hospital Cosme Argerich, Buenos Aires, Argentina; 2 Virology Unit, Hospital Francisco Muñiz, Buenos Aires, Argentina; 3 Neonatology Unit, Hospital Cosme Argerich, Buenos Aires, Argentina

## Abstract

**Introduction:**

Surveillance of primary resistance to antiretroviral drugs is particularly important in pregnant population, in which infection by drug-resistant HIV has not only implications for maternal treatment, but could also jeopardize the efficacy of neonatal prophylaxis. We aim to describe the prevalence of resistance associated mutations (RAMs) in pregnant women with intrapartum HIV diagnosis in a public hospital of Buenos Aires, Argentina.

**Materials and Methods:**

Prospective pilot study (period from 2008 to October 2013). Plasma samples were tested for viral load by Versant HIV-1 RNA 3.0 (bDNA) and sequenced using HIV-1 TRUGENE™Genotyping Kit (Siemens). The prevalence of RAMs was analyzed according to World Health Organization (WHO) criteria.

**Results:**

Of 231 HIV-infected pregnant women assisted, 6% (n=14) had intrapartum diagnosis of HIV infection. 12 patients (85.7%) had previous pregnancies, 10 (71.4%) had inadequate prenatal care and 3 (23.1%) seroconverted during pregnancy. Maternal characteristics (expressed medians and ranges) were: age 25.5 (16–35) years; gestational age at birth: 39 (30–42) weeks; CD4 count: 500 (132–925) cells/µL; viral load: 9418 (1800–55299) copies/mL. No one had hepatitis B virus (HBV) or hepatitis C virus (HCV) coinfection; four (33.3%) had syphilis. Eight patients (57.1%) had vaginal delivery and six emergency C-section (42.9%). In six cases (46.2%), membrane rupture was spontaneous; four patients (28.6%) failed to receive intrapartum zidovudine (ZDV) infusion. In 12 patients a genotypic resistance test was performed: two (16.7%) had WHO RAMs corresponding to K103N mutation in both cases, conferring high-level resistance to nevirapine (NVP) and efavirenz. Two newborns (14.3%) were preterm. All received neonatal prophylaxis: ZDV in 1 case and combined prophylaxis (ZDV/3TC/NVP) in the remaining 13 (92.9%). All newborns were formula-fed. Two (14.3%) had congenital syphilis, one of whom died. One newborn was HIV-infected (positive proviral DNA at 24 hours of life, wild-type HIV).

**Conclusions:**

This pilot study suggests that levels of transmitted resistance in this high-risk population of pregnant women could be moderate to high. We preliminarily observed high-level resistance to NVP: if this finding is confirmed with a larger sample, it could potentially jeopardize the utility of this drug in the combined neonatal prophylaxis recommended in the absence of maternal antiretroviral therapy.
